# A History of Abuse and Operative Delivery – Results from a European Multi-Country Cohort Study

**DOI:** 10.1371/journal.pone.0087579

**Published:** 2014-01-31

**Authors:** Berit Schei, Mirjam Lukasse, Elsa Lena Ryding, Jacquelyn Campbell, Helle Karro, Hildur Kristjansdottir, Made Laanpere, Anne-Mette Schroll, Ann Tabor, Marleen Temmerman, An-Sofie Van Parys, Anne-Marie Wangel, Thora Steingrimsdottir

**Affiliations:** 1 Department of Public Health and General Practice, Norwegian University of Science and Technology, Trondheim, Norway; 2 Department of Obstetrics and Gynaecology, St.Olav's University Hospital, Trondheim, Norway; 3 Department of Health, Nutrition and Management, Oslo and Akershus University College of Applied Sciences, Oslo, Norway; 4 Department of Women's and Children's Health, Division of Obstetrics and Gynaecology, Karolinska Institutet/University Hospital, Stockholm, Sweden; 5 John Hopkins University, School of Nursing, Baltimore, Maryland, United States of America; 6 Department of Obstetrics and Gynaecology, University of Tartu, Tartu, Estonia; 7 Department of Obstetrics and Gynaecology, Landspitali University Hospital, Reykjavik, Iceland; 8 Directorate of Health, Reykjavik, Iceland; 9 Centre of Fetal Medicine, Department of Obstetrics, Copenhagen University Hospital, Rigshospitalet, Copenhagen, Denmark; 10 Faculty of Health Sciences, University of Copenhagen, Copenhagen, Denmark; 11 Department of Obstetrics and Gynaecology, Ghent University Hospital, Ghent, Belgium; 12 Malmö University, Faculty of Health and Society, Malmö, Sweden; 13 Primary Health Care of the Capital Area, Centre of Development, Reykjavik, Iceland; Baylor College of Medicine, United States of America

## Abstract

**Objective:**

The main aim of this study was to assess whether a history of abuse, reported during pregnancy, was associated with an operative delivery. Secondly, we assessed if the association varied according to the type of abuse and if the reported abuse had been experienced as a child or an adult.

**Design:**

The Bidens study, a cohort study in six European countries (**B**elgium, **I**celand, **D**enmark, **E**stonia, **N**orway, and **S**weden) recruited 6724 pregnant women attending routine antenatal care. History of abuse was assessed through questionnaire and linked to obstetric information from hospital records. The main outcome measure was operative delivery as a dichotomous variable, and categorized as an elective caesarean section (CS), or an operative vaginal birth, or an emergency CS. Non-obstetrically indicated were CSs performed on request or for psychological reasons without another medical reason. Binary and multinomial regression analysis were used to assess the associations.

**Results:**

Among 3308 primiparous women, sexual abuse as an adult (≥18 years) increased the risk of an elective CS, Adjusted Odds Ratio 2.12 (1.28–3.49), and the likelihood for a non-obstetrically indicated CS, OR 3.74 (1.24–11.24). Women expressing current suffering from the reported adult sexual abuse had the highest risk for an elective CS, AOR 4.07 (1.46–11.3). Neither physical abuse (in adulthood or childhood <18 years), nor sexual abuse in childhood increased the risk of any operative delivery among primiparous women. Among 3416 multiparous women, neither sexual, nor emotional abuse was significantly associated with any kind of operative delivery, while physical abuse had an increased AOR for emergency CS of 1.51 (1.05–2.19).

**Conclusion:**

Sexual abuse as an adult increases the risk of an elective CS among women with no prior birth experience, in particular for non-obstetrical reasons. Among multiparous women, a history of physical abuse increases the risk of an emergency CS.

## Introduction

Interventions during childbirth, such as operative delivery, aim to ensure best possible health for the mother and child. There are interventions that are obviously necessary and life-saving for both mother and child [1]. However, sometimes the indication for intervention is subject to debate and even considered non-medical, such as caesarean section (CS) performed for fear of childbirth or on maternal request only [2], [3].

Violence against women (VAW) is a broad term covering a large range of traumatic events and abuse occurring during a woman's life-span. VAW has been shown to increase the risk for complications during pregnancy and may thereby increase interventions during delivery [4]–[8]. VAW also increases the risk of psychological distress and fear of childbirth, which in turn may become the indication for CS on maternal request [9]–[11]. Furthermore, VAW may alter a woman's stress response and affect ability to communicate with obstetric staff during labor, thus increasing the risk of interventions [12]. Few studies exists which examine the association between abuse and mode of delivery. The studies that exist are limited in terms of the type of abuse addressed and inconclusive in their findings [13]–[18]. Two studies show a major increase in risk of CS for women who have been subjected to rape in adulthood [15], [18]. However, the women included were referred to a mental health team for known psychological problems and because of their desire to give birth by elective CS [15], [18]. The association between abuse and mode of delivery may not be the same among women attending routine antenatal care. The aim of this study was to test the a priori hypothesis that a history of abuse is associated with an increased risk of operative delivery, i.e. a caesarean section (elective or emergency) or an operative vaginal delivery, among women attending routine antenatal care. Secondly, we wanted to assess if potential associations varied between types of abuse (emotional, physical or sexual), and whether the abuse had occurred during childhood or adulthood. Thirdly, where an association between a type of abuse and CS was found, we explored the association with non-obstetric indication.

## Methods

The Bidens study, a six-country (Belgium, Iceland, Denmark, Estonia, Norway, and Sweden) cohort study of unselected pregnant women, was the result of an EU-funded collaboration between the Norwegian University of Science and Technology (NTNU) and partners from Universities and Hospitals in six European countries. A short description of the study sites is given in Table 1.

**Table 1:** Presentation of recruitment sites in the Bidens study.

Country	City	Characteristics (inhabitants)	Recruitment Site	Number of deliveries in 2008	Number of deliveries in geographical area
**Belgium**	Gent	Third largest city (240.000)	Gent University Hospital	1217	6674
	Waregem	(36.000)	Onze Lieve Vrouw van Lourdes/‘Our Lady from Lourdes’	721	721
	Ieper	(35.000)	Jan Yperman Hospital	1091	1091
**Iceland**	Reykjavik	Capital (200.000)	Landspitali University Hospital	3373	4118
**Denmark**	Copenhagen	Capital (1.811.239)	University Hospital	3730	21101§
**Estonia**	Tallinn	Capital (400 000)	West-Tallinn Central Hospital	3283	7421
			East-Tallinn Central Hospital	4386	
	Tartu	Second largest city in Estonia (100 000)	Tartu University Hospital	2325	1994
	Kohtla-Järve	North-East Estonia (46 000) 80% Russian speaking	East-Viru Central Hospital	515	1490
**Norway**	Drammen	(60 000)	Buskerud Regional Hospital	1961	3003*
	Oslo	Capital (560 000)	Rikshospitalet, OUS	2238	10252*
	Tromsø	Most northern city (67 000)	University hospital in North -Norway	1509	1961*
	Trondheim	(165 000)	St.Olavs University hospital	3483	3830*
	Ålesund	(42 000)	Hospital in Ålesund	1291	2813*
**Sweden**	Malmö	(295 000)	Antenatal Care Clinics (ANC):	University Hospital MAS 4359	Selected ANC represent Approx. 60% of all births of the catchments area

References for number of deliveries in the geographical area: Belgium: SPE (Studiecentrum Perinatale Epidemiologie) 2008. Iceland: The Icelandic Birth register for 2008. Denmark: http://www.sst.dk/Indberetning%20og%20statistik/Sundhedsdata/Foedsler_fertilitetsbehandling_og_abort/foedsler1.aspx.

§ Born at hospital Estonia: http://www.tai.ee/et/tegevused/registrid/meditsiiniline-sunniregister-ja-raseduskatkestus-andmekogu/statistika; Estonian Medical Birth Registry.

Sweden regional data: http://www.socialstyrelsen.se/register/halsodataregister/medicinskafodelseregistret Norway: Medical Birth Registry.

* Number of newborn ≥22 weeks gestation.

### Participants

Recruitment took place between March 2008 and August 2010. Women who consented subsequently completed a questionnaire and allowed the extraction of specified data on their delivery from their medical notes. Due to country specific organization as well as requirements of local ethical committees, minor variations in the recruitment procedure occurred.

In *Belgium*, women were approached by the midwife or secretary when attending antenatal care. Consenting women were asked to complete the questionnaire in a separate room. In *Iceland* women were recruited when attending routine ultrasound and returned completed forms by mail. In *Denmark*, women were given information about the study when attending early routine ultrasound screening and were mailed the questionnaire later. They returned the questionnaire by mail or when attending their next ultrasound examination. In *Estonia*, women were invited to participate while visiting for an antenatal consultation. After completing the questionnaire, it was left in a mailbox at the clinic. In *Norway* women, after attending routine ultrasound, received the questionnaire by mail and returned it by mail. Non-responders were send one reminder. In *Sweden*, the questionnaire was administered to women when attending routine glucose tolerance test and filled out during the two hours gap between the blood samplings. Belgium and Sweden were not permitted to record non-participation. The estimated response rate varied between 50% in Norway to 90% in Estonia.

### Inclusion and exclusion criteria

All women required sufficient language skills to fill out the form. In Estonia, women could choose to fill out an Estonian or Russian language questionnaire. In Belgium, Iceland and Denmark women less than 18 years of age were excluded. In Denmark, only women from the local geographical area were invited. In Belgium, women who could not be separated from their accompanying person were not recruited. In Iceland, Denmark and Norway, women with major fetal pathology were excluded from the study.

#### Sample size for this study

Of the 7200, women who consented and returned the questionnaire 6724 were included in this study. Of the 476 excluded, 47 had failed to answer two or more of the questions on abuse, 304 lacked information about mode of delivery, 122 lacked parity, and the pregnancy of three women ended before 22 weeks gestation.

### Questionnaire

**Figure 1:**
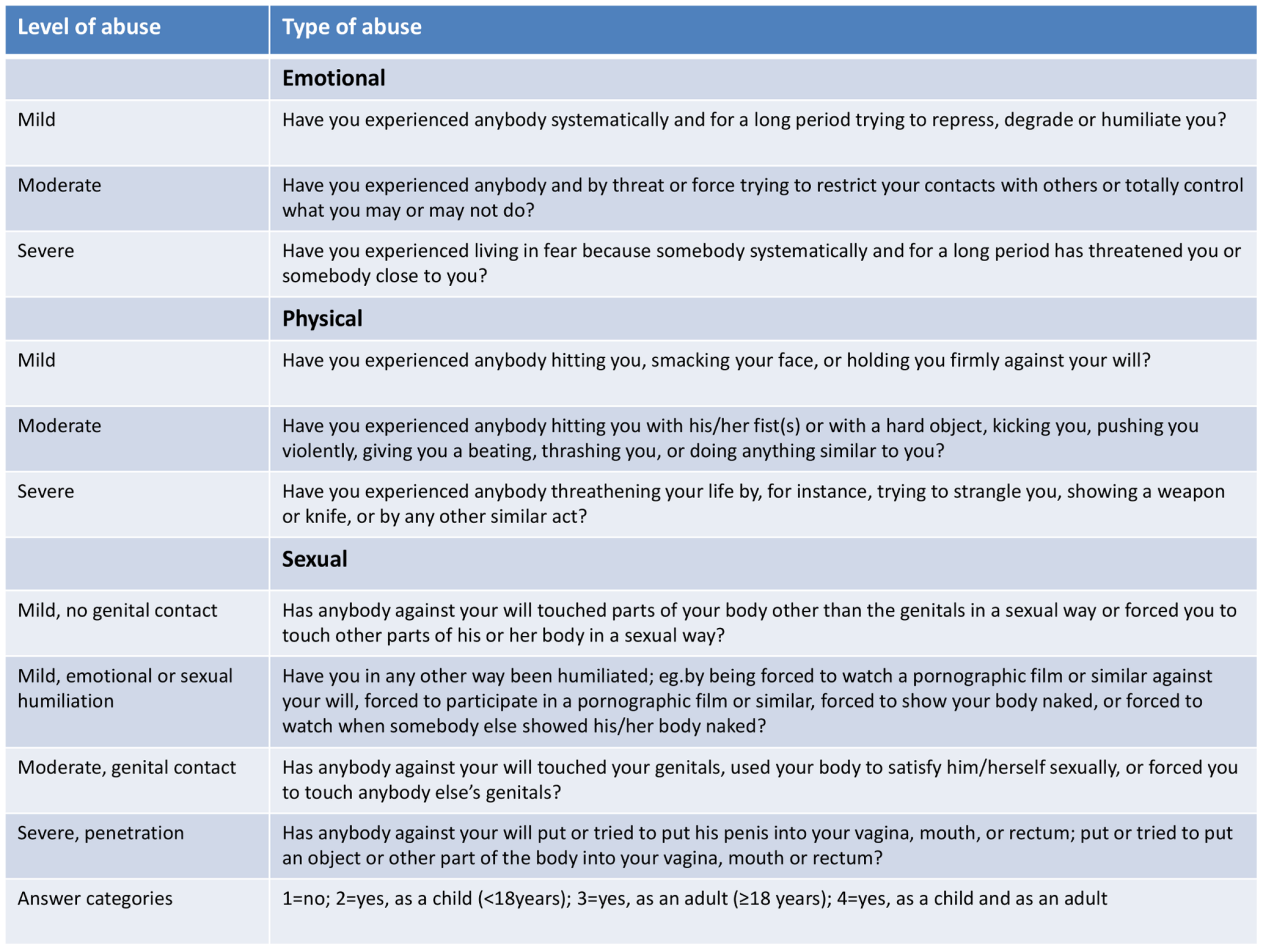
The Norvold Abuse Questionnaire (NorAQ) questions on emotional, physical and sexual abuse.

The main instrument of the present study is a 68 items questionnaire, partly based on the NorAQ (Norvold Abuse Questionnaire, Figure 1), which was developed in a previous multi-centre study among gynaecological patients in the Nordic countries [19]. The different types of abuse and severity of abuse were defined in NorAQ by a validated set of thirteen descriptive questions [20]. Also included in the questionnaire were questions on post-traumatic stress symptoms [21], fear of childbirth, using the Wijma Delivery Expectancy Questionnaire (W-DEQ) [22], and a short version of the Edinburgh Postpartum Depression Scale (EPDS-5) [23]. Method and experience of previous deliveries, as well as preference of mode of delivery, were assessed. A complete version of the questionnaire was developed in English. Where a previously translated version of the NorAQ, W-DEQ or EPDS was available, this was used. Otherwise, the questionnaire was translated into the required languages by a native speaker (Flemish, Icelandic, Danish, Estonian, Russian, Norwegian and Swedish) and then translated back again into the source language. The original and back-translated versions were used to determine the final version.

### Follow-up

Birth outcome data was collected from hospital records and recorded on the outcome sheet prepared for this study.

### Variables

Main exposure: Emotional, physical and sexual abuse was assessed in three identically structured sections [19]. For each type and level of abuse, the answer categories were no, yes as a child, yes as an adult, or yes both as a child and as an adult and classified according to the most severe level reported (mild, moderate and, severe). Women were asked if they experienced the indicated abuse during the past 12 months, which was coded as recent. The degree of current suffering was measured on a visual analogous scale (0–10) and recoded into zero for values 0–4 and 1 for values 5–10. Women were defined as having experienced any abuse if they answered yes to at least one of the questions of sexual, emotional and physical abuse, except mild physical abuse, which showed low specificity in the validation study [20].

Education was coded into four levels: primary school (9 years), secondary school (13 years), higher education (university or college), <4 years and ≥4 years. Women were considered to have a twin pregnancy if they had reported this in the questionnaire.

The outcome, operative delivery was defined as a dichotomous yes or no variable to test the main hypothesis for this study, and as a categorical variable with the following categories: 0) spontaneous vaginal 1) elective Caesarean Section (CS) 2) forceps or vacuum extraction and 3) emergency CS. Indications options on the outcome sheet for operative deliveries included: fetal distress, dystocia, breech, maternal exhaustion, maternal request, psychosocial indications, other obstetrical indications, and unknown. A CS was defined as non-obstetrically indicated when “maternal request” or “psychosocial indication” were the reported reasons without another medical indication. The indication for the operative delivery was taken from the hospital record, as was gestational age (based on ultrasound during pregnancy) at birth.

### Ethical considerations

The study was conducted in accordance with the ethical guidelines developed by WHO [24]. The information letter instructed women to complete the form in a place where they could be undisturbed and included telephone numbers and e-mail addresses to contact if needed. Additionally, in Belgium, Estonia and Sweden the participants had the opportunity to complete the questionnaires at the clinic, and measures were installed to avoid accompanying persons to be with them. Formal approvals of local ethical committees and data protection agencies were obtained at all sites, as listed below.

*Belgium:* The Ethical Committee of Ghent University acted as the central ethical committee for the study; U(Z) Gent, 22012008/B67020072813, date of approval: 1^st^ February 2008, Waregem hospital date added: 21^st^ October 2008.

*Iceland:* The scientific board approved the study (24.06.2008-*VSN-b2008030024/03-15*) according to Icelandic regulations, date: 24^th^ June 2008.

In *Denmark*, even though ethical approval for non-invasive studies is not required, the study was presented to the Research Ethics Committee of the Capital Region, who found no objections to the study (H-A-2008-002), date: 11^th^ February 2008. Permission was obtained from the Danish Data Protection Agency (J.nr. 2007-41-1663).

In *Estonia*, ethical permission was given by the Ethics Review Committee on Human Research of the University of Tartu, Estonia; 190/M-29, 192/-22, 196/X-2, date: 17^th^ December 2007, East-Tallinn Central Hospital added: 19^th^ January 2009, Russian language and prolonged period added: 22^nd^ February 2010, East-Viru Central Hospital added: 26^th^ April 2010.

In *Norway*, the Regional Committee for Medical Research Ethics in North approved the study (72/2006), date: 29^th^ August 2007; and the Data Inspectorate (NSD) (15214/3/) also approved the study, date: 19^th^ December 2007.

In *Sweden*, the study was approved by the Regional Ethical Committee in Stockholm (2006/354-31/1), date: 14^th^ June 2006.

The data was anonymised prior to analysis.

### Statistical analyses

Power calculation was based on the main hypotheses that exposure to any abuse increased the risk of any operative delivery, OR 1.4 (α = 0.05, β = 0.20), assuming one exposed and four non-exposed within an unselected cohort of pregnant women [19], [25]. In total, 2500 women needed to be recruited, allowing for stratified analysis based on parity approximately 5000.

Cross-tabulation was used to quantify socio-demographic, life-style and obstetric characteristics factors by country of residence and mode of delivery. Frequency analyses were used to quantify the prevalence of the different types of abuse by category, age <18 or ≥18, level of severity, current suffering and recentness. All regression analyses were stratified for parity. The main hypothesis was tested by binary logistic regression analysis. The association between different abuse categories and the different kinds of operative delivery were analysed by multinomial regression analyses. Based on the literature and our experience, we included all of the variables (which we had information on) correlated with operative delivery (outcome) and, for each of them evaluated if they were likely to be the result of the exposure (abuse). Of the factors influencing operative delivery we considered the following as correlated to but not the result of the exposure: age, twin pregnancy, gestational age at birth, and country of residence. These variables were included in the adjusted regression analyses. Smoking status [25], [26], alcohol consumption [26], [27], use of epidural analgesia [14], [18], birth weight [26], [27] could be the result of the independent variable (abuse) and were not included in the model. Factors such as depressive symptoms, post-traumatic stress symptoms and fear of childbirth were also excluded from the model as we considered them to fall on the pathway between exposure (abuse) and operative delivery. As a previous CS could be the result of abuse before the related pregnancy, we did not want to enter it into all the analyses, but estimated its impact on the significant association(s) found for multiparous women. We estimated crude and adjusted odd ratios (AORs) and 95% confidence intervals (CIs). All analyses were two-sided at α = 0.05. The comparison group for all analyses was women without a reported history of abuse. The statistical program used was PASW 20.

## Results

Among the 6724 women in our study, 2323 (34.5%) reported having experienced any abuse (of any type) at some point in their lives, 1567 (23.3%) as a child, 1309 (19.5%) as an adult and 553 (8.2%) both as a child and an adult. The distribution of responses between countries, for socio-demographic and obstetrical characteristics are presented in Table 2. The participants in Denmark (both primiparous and multiparous women) were older than women elsewhere, while the youngest primiparous women were in Iceland and multiparous in Belgium. The caesarean section (CS) rate was highest in Denmark and lowest in Sweden. Birth weight below 2500 g was highest in Denmark and birth weight ≥4000 g was most common in Iceland and least common in Belgium. Socio-demographic and obstetric characteristics by mode of delivery can be found in table 3.

**Table 2:** Socio-demographic and obstetric characteristics of women in the Bidens study N = 6724.

	Belgium n = 819	Iceland n = 585	Denmark n = 1268	Estonia n = 874	Norway n = 2234	Sweden n = 944	Total N = 6724
**Age** mean (SD)	28.4 (4.3)	29.7 (5.0)	31.9 (4.0)	28.2 (5.1)	30.5 (5.0)	30.4 (4.6)	30.1 (4.9
Primiparous mean (SD)	27.2 (4.0)	26.8 (4.4)	30.7 (4.0)	25.5 (4.2)	28.6 (4.9)	29.1 (4.4)	28.4 (4.7)
Multiparous mean (SD)	29.8 (4.2)	31.6 (4.5)	33.4 (3.6)	30.5 (4.7)	32.0 (4.4)	32.1 (4.4)	31.7 (4.4)

	n (%)	n (%)	n (%)	n (%)	n (%)	n (%)	n (%)
**Education**							
Primary (6–9 years)	13 (1.6)	42 (7.2)	19 (1.5)	69 (7.9)	53 (2.4)	31 (3.3)	227 (3.4)
Secondary (10–13 years)	301 (36.8)	141 (24.1)	118 (9.3)	286 (32.7)	568 (25.4)	278 (29.4)	1692 (25.2)
College/University <4 year	338 (41.39	184 (31.5)	460 (36.3)	242 (27.7)	688 (30.8)	221 (23.4)	2133 (31.7)
College/University ≥4 years	161 (19.7)	215 (36.8)	660 (52.1)	270 (30.9)	913 (40.9)	401 (42.5)	2620 (39.0)
Missing	6 (0.7)	3 (0.5)	11 (0.9)	7 (0.8)	12 (0.5)	13 (1.4)	52 (0.8)
**Civil status**							
Married/Cohabiting	784 (95.7)	542 (92.6)	1206 (95.6)	828 (94.7)	2144 (96.0)	904 (95.8)	6408 (95.3)
Other	25 (3.19	39 (6.7)	49 (3.9)	45 (5.1)	77 (3.4)	37 (3.9)	272 (4.0)
Missing	10 (1.29	4 (0.7)	13 (1.0)	1 (0.1)	13 (0.6)	3 (0.3)	44 (0.7)
**Parity**							
Multiparous	380 (46.4)	356 (60.9)	535 (42.2)	469 (53.5)	1276 (57.1)	401 (42.5)	3416 (50.8)
Primiparous	439 (53.6)	229 (39.1)	733 (57.8)	406 (46.5)	958 (42.9)	543 (57.5)	3308 (49.2)
**Gestational age at birth**							
<37 weeks	42 (5.1)	24 (4.1)	73 (5.8)	46 (5.3)	121 (5.4)	55 (5.8)	361 (5.4)
≥37 weeks	776 (94.7)	561 (95.6)	1194 (94.2)	826 (94.5)	2102 (94.1)	888 (94.1)	6347 (94.4)
Missing	1 (0.1)	0	1 (0.1)	2 (0.2)	11 (0.5)	1 (0.1)	16 (0.2)
**Induced**	192 (23.4)	117 (20.0)	245 (19.3)	112 (12.8)	408 (18.3)	83 (8.8)	1157 (17.2)
**Epidural Analgesia**	552 (67.4)	197 (33.7)	387 (30.5)	112 (12.8)	585 (26.2)	181 (19.2)	2014 (30.0)
**Mode of delivery**							
Spontaneous vaginal	590 (72.0)	439 (75.0)	895 (70.6)	679 (77.7)	1696 (75.9)	735 (77.9)	5034 (74.9)
Operative delivery	229 (28.0)	146 (25.0)	373 (29.4)	195 (22.3)	538 (24.1)	209 (22.1)	1690 (25.1)
Elective CS	71 (8.7)	40 (6.8)	138 (10.9)	63 (7.2)	171 (7.7)	59 (6.3)	542 (8.1)
Operative vaginal	98 (12.0)	57 (9.7)	117 (9.2)	50 (5.7)	189 (8.5)	69 (7.3)	580 (8.6)
Emergency CS	60 (7.3)	49 (8.4)	118 (9.3)	82 (9.4)	178 (8.0)	81 (8.6)	568 (8.4)
**Birth weight**							
<2500 g	25 (3.1)	17 (2.9)	61 (4.8)	30 (3.4)	80 (3.6)	30 (3.2)	243 (3.6)
2500–4000 g	733 (89.5)	410 (70.1)	1003 (79.1)	680 (77.8)	1756 (78.8)	728 (77.1)	5310 (79.0)
≥4000 g	59 (7.2)	158 (27.0)	196 (15.5)	162 (18.5)	391 (17.5)	183 (19.4)	1149 (17.1)
Missing	2 80.2)	0	8 (0.6)	2 (0.2)	7 (0.3)	3 (0.3)	22 (0.3)
**Apgar score**							
<7 after 5 min.	7 (0.9)	18 (3.1)	4 (0.3)	9 (1.0)	19 (0.9)	17 (1.8)	74 (1.1)
≥7 after 5 min.	804 (98.2)	562 (96.1)	1253 (98.8)	860 (98.4)	2199 (98.4)	910 (96.4)	6588 (98.0)
Missing	8 (1.0)	5 (0.9)	11 (0.9)	5 (0.6)	16 (0.7)	17 (1.8)	62 (0.9)
**Twins**	19 (2.3)	5 (0.9)	45 (3.5)	14 (1.6)	53 (2.4)	20 (2.1)	156 (2.3)

**Table 3:** Socio-demographic and obstetric characteristics of women in the Bidens study (N = 6724) by mode of delivery.

	Elective CS n = 542	Spontaneous vaginal n = 5034	Operative vaginal n = 580	Emergency CS n = 568
**Age**: mean (SD)	31.9 (5.0)	30.0 (4.8)	29.5 (4.7)	30.1 (5.1)
Primiparous	30.1 (5.3)	28.1 (4.6)	29.1 (4.6)	29.1 (4.9)
Multiparous	32.9 (4.5)	31.6 (4.4)	31.2 (4.6)	32.5 (4.6)

	n (%)	n (%)	n (%)	n (%)
**Education**				
Primary (6–9 years)	11 (2.1)	182 (3.6)	12 (2.1)	22 (3.9)
Secondary (10–13 years)	135 (25.3)	1275 (25.5)	140 (24.4)	142 (25.2)
College/University <4 year	168 (31.5)	1574 (31.5)	188 (32.8)	203 (36.1)
College/University ≥4 years	219 (41.1)	1972 (39.4)	233 (40.7)	196 (34.8)
**Civil status:**				
Married/Cohabiting	518 (96.3)	4800 (95.9)	552 (96.3)	538 (95.2)
Other	20 (3.7)	204 (4.1)	21 (3.7)	27 (4.8)
**Parity:**				
Multiparous	338 (62.4)	2789 (55.4)	115 (19.8)	174 (30.6)
Primiparous	204 (37.6)	2245 (44.6)	465 (80.5)	394 (69.4)
**Gestational age at birth**				
<37 weeks	57 (10.5)	196 (3.9)	22 (3.8)	86 (15.1)
≥37 weeks	485 (89.5)	4838 (96.1)	558 (96.2)	482 (84.9)
**Induced**	13 (2.4)	813 (16.2)	143 (24.7)	188 (33.1)
**Epidural Analgesia**	67 (12.4)	1322 (26.3)	342 (59.0)	283 (49.8)
**Birth weight:**				
<2500 g	45 (8.3)	114 (2.3)	12 (2.1)	72 (12.7)
2500–4000 g	478 (88.4)	4746 (94.6)	552 (95.8)	459 (80.8)
≥4000 g	18 (3.3)	157 (3.1)	12 (2.1)	37 (6.5)
**Apgar score:**				
<7 after 5 min	4 (0.7)	34 (0.7)	20 (3.5)	16 (2.8)
≥7 after 5 min.	537 (99.3)	4948 (99.3)	556 (96.5)	547 (97.2)
**Twins**	43 (8.1)	74 (1.5)	17 (3.0)	22 (3.9)

A history of any abuse was not associated with any operative delivery (as a dichotomous variable), neither among primiparous or multiparous women delivery, AOR 1.16 (0.99–1.36) and AOR 1.04 (0.86–1.25) respectively (not in the tables). Among multiparous women, only a history of physical abuse was associated with a significant increase in emergency CSs (Table 4), AOR 1.51 (1.05–2.19). This association was attenuated when we added previous CS as a covariate into the analysis, OR 1.48 (1.001–2.18). Of the 512 (15.0%) multiparous women who had a CS, 76 (14%) had a non-obstetrically indicated CS. No significant association between a history of physical abuse and non-obstetrically indicated CS was observed, crude OR 0.94 (0.52–1.70). The most common reason for emergency CS among multiparous women with a history of physical abuse was fetal distress, followed by other medical reasons and dystocia. Table 5 presents different types of childhood and adult abuse by operative delivery among primiparous women. Women with no prior birth experience who reported a history of adult sexual abuse were significantly more likely to be delivered by elective CS, AOR 2.12 (1.28–3.49). This likelihood increased when they in addition reported either physical or emotional abuse experienced as an adult (Table 6). Primiparous women expressing current suffering from the reported adult sexual abuse had the highest risk for an elective CS, AOR 4.07 (1.46–11.3). Of all the primiparous women, 18% (598) were delivered by CS. Of these, 42 (7.0%) had a non-medical indication. Adult sexual abuse increased the odds of a CS without a medical indication, OR 3.74 (1.24–11.24). The most common indication for an elective CS among primiparous women who reported adult sexual abuse was a breech presentation, followed by other medical reasons and maternal request.

**Table 4:** The association between (Adjusted OR*) different types of abuse and operative delivery for multiparous women (n = 3416) in the Bidens study.

	Abuse	Elective CS	Elective CS	Operative vaginal	Operative vaginal	Emergency CS	Emergency CS
	n (%)	%	OR (95% CI)	%	OR (95% CI)	%	OR (95% CI)
**No abuse**	2214 (64.8)	9.8	1	3.6	1	4.9	1
**Any abuse**	1202 (35.2)	10.1	1.05 (0.83–1.33)	3.0	0.83 (0.56–1.23)	5.4	1.11 (0.80–1.53)
**Any abuse <18**	797 (23.3)	10.3	1.08 (0.82–1.41)	3.1	0.88 (0.55–1.39)	5.5	1.13 (0.78–1.64)
**Any abuse ≥18**	704 (20.6)	9.7	0.98 (0.73–1.31)	2.8	0.79 (0.48–1.31)	5.0	1.00 (0.67–1.49)
**Emotional abuse**	638 (18.7)	9.7	1.00 (0.74–1.35)	2.2	0.61 (0.34–1.09)	6.3	1.30 (0.89–1.91)
**Emotional abuse <18**	385 (11.3)	10.6	1.12 (0.78–1.61)	2.3	0.66 (0.33–1.33)	6.2	1.31 (0.82–2.10)
**Emotional abuse ≥18**	369 (10.8)	8.7	0.86 (0.58–1.28)	2.4	0.67 (0.33–1.36)	5.7	1.15 (0.70–1.87)
**Physical abuse**	653 (19.1)	11.3	1.24 (0.93–1.65)	3.5	1.03 (0.64–1.66)	6.9	1.51 (1.05–2.19)
**Physical abuse <18**	328 (9.6)	11.6	1.28 (0.88–1.86)	3.0	0.89 (0.46–1.75)	7.3	1.57 (0.97–2.52)
**Physical abuse ≥18**	383 (11.2)	10.7	1.13 (0.79–1.62)	3.4	0.99 (0.54–1.80)	6.0	1.30 (0.81–2.08)
**Sexual abuse**	561 (16.4)	10.5	1.09 (0.80–1.49)	2.7	0.74 (0.42–1.30)	4.8	0.97 (0.62–1.51)
**Sexual abuse <18**	398 (11.6)	10.1	1.05 (0.73–1.50)	2.8	0.77 (0.40–1.46)	4.8	0.96 (0.58–1.60)
**Sexual abuse ≥18**	221 (6.5)	11.3	1.14 (0.73–1.78)	2.7	0.77 (0.33–1.80)	4.5	0.91 (0.46–1.79)

* Adjusted for age, twin pregnancy, gestational age less than 37 weeks, and country of residence.

Compared to women not reporting any abuse.

**Table 5:** The association (Adjusted OR*) between different types of abuse and mode of delivery for primiparous women (n = 3308) in the Bidens study.

	Abuse	Elective CS	Elective CS	Operative vaginal	Operative vaginal	Emergency CS	Emergency CS
	n (%)	%	OR (95% CI)	%	OR (95% CI)	%	OR (95% CI)
**No abuse**	2187 (66.1)	5.9	1	13.8	1	11.5	1
**Any abuse**	1121 (33.9)	6.7	1.22 (0.90–1.66)	14.5	1.11 (0.97–1.37)	12.7	1.16 (0.92–1.45)
Any abuse <18	770 (23.3)	5.6	1.04 (0.72–1.51)	14.7	1.13 (0.89–1.44)	13.0	1.21 (0.94–1.56)
Any abuse ≥18	605 (18.3)	8.6	1.45 (1.02–2.06)	13.2	0.97 (0.74–1.27)	12.6	1.09 (0.82–1.45)
**Emotional abuse**	623 (18.8)	7.4	1.38 (0.96–1.98)	15.1	1.21 (0.93–1.57)	13.3	1.24 (0.94–1.64)
Emotional abuse <18	422 (12.7)	6.6	1.28 (0.82–1.98)	14.2	1.15 (0.84–1.56)	14.2	1.37 (1.00–1.88)
Emotional abuse ≥18	316 (9.5)	8.5	1.50 (0.96–2.36)	13.9	1.07 (0.76–1.53)	13.3	1.17 (0.82–1.69)
**Physical abuse**	566 (17.1)	6.7	1.21 (0.82–1.78)	14.1	1.03 (0.78–1.36)	10.2	0.93 (0.68–1.27)
Physical abuse <18	339 (10.2)	5.6	1.05 (0.63–1.76)	14.2	1.07 (0.76–1.50)	10.6	1.00 (0.69–1.47)
Physical abuse ≥18	297 (9.0)	8.8	1.45 (0.92–2.30)	13.8	0.97 (0.68–1.40)	9.4	0.81 (0.53–1.23)
**Sexual abuse**	495 (15.0)	7.7	1.42 (0.96–2.10)	14.1	1.07 (0.80–1.43)	12.7	1.19 (0.88–1.61)
Sexual abuse <18	342 (10.3)	5.5	1.00 (0.60–1.67)	12.9	0.93 (0.66–1.32)	12.0	1.07 (0.75–1.54)
Sexual abuse ≥18	200 (6.0)	11.0	2.12 (1.28–3.49)	16.0	1.31 (0.87–1.98)	14.0	1.39 (0.90–2.16)

* Adjusted for age, twin pregnancy, gestational age less than 37 weeks, and country of residence.

Compared to women not reporting any abuse.

**Table 6:** The association between adult sexual abuse and mode of delivery for primiparous women (n = 3308) in the Bidens study.

		Elective CS		Operative vaginal delivery		Emergency CS	
Exposure	n	%	Adjusted OR (95% CI)	%	Adjusted OR (95% CI)	%	Adjusted OR (95% CI)
No abuse	2187	5.9	1	13.8	1	11.5	1
**Any adult sexual abuse** *	200	11.0	2.12 (1.28–3.49)	16.0	1.31 (0.87–1.98)	14.0	1.39 (0.90–2.16)
**Adult sexual abuse** *							
mild	46	15.2	2.21 (0.93–5.26)	6.5	0.42 (0.13–1.38)	8.7	0.69 (0.24–1.99)
moderate	41	9.8	1.77 (0.57–5.52)	19.5	1.72 (0.75–3.92)	17.1	1.83 (0.76–4.40)
severe	113	9.7	2.15 (1.10–4.23)	18.6	1.66 (1.00–2.77)	15.0	1.60 (0.91–2.79)
**Adult sexual abuse and:**							
adult physical*	70	12.9	2.88 (1.34–6.19)	17.1	1.52 (0.78–2.95)	14.3	1.61 (0.79–3.30)
adult emotional*	59	13.6	2.93 (1.30–6.60)	20.3	1.84 (0.93–3.63)	11.9	1.27 (0.55–2.93)
adult emotional and physical*	38	10.5	2.69 (0.88–8.21)	23.7	2.34 (1.04–5.26)	15.8	2.02 (0.79–5.16)
current suffering*	37	13.5	4.07 (1.46–11.3)	16.2	1.86 (0.72–4.74)	21.6	2.97 (1.26–6.98)
child sexual abuse*	47	6.4	1.09 (0.32–3.69)	12.8	0.93 (0.38–2.27)	12.8	1.08 (0.44–2.65)
recent experience†	12	16.7	3.52 (0.73–16.8)	0	0	8.3	0.82 (0.10–6.48)

* Adjusted age, twin pregnancy, gestational age less than 37 weeks, and country of residence.

† Due to few cases adjusted only for age.

Compared to women not reporting any abuse.

## Discussion

In our multi-country study of pregnant women attending routine antenatal care, followed through to delivery, the main hypothesis that a history of any abuse was associated with an operative delivery was not confirmed. Rather, among multiparous women, a reported history of physical abuse was the only type of abuse associated with operative delivery, as it increased the odds for emergency caesarean section. Among primiparous women, only a history of adult sexual abuse was associated with operative delivery, through an increase in elective CS. Primiparous women with a history of adult sexual abuse were more likely to have a non-obstetrical indication when delivered by CS. In addition was current suffering from adult sexual abuse associated with an increased risk for an emergency CS.

A great strength of our study is that it is based on pregnant women attending routine antenatal care. The recruitment procedure varied across sites, both for the invitation to participate, where the questionnaire was completed and how it was returned. This may have introduced information bias. The participation rates varied between countries, but the background characteristics did not indicate any significant selection bias when compared to information from official health authorities. Guidelines for antenatal care and interventions during delivery vary between the participating countries. For example, neither in Belgium nor in Estonia is maternal request alone an accepted indication for medical interventions such as delivery by CS. The uniform results across the participating countries strengthen our finding that among primiparous women a history of sexual abuse as an adult increases the risk for elective CS, regardless of maternal request being an acceptable indication. At all the sites included in the study, epidural analgesia during labour is available, however the rates of use varied greatly between the countries. We do not know how this may influence our results.

Victims of adult sexual abuse with posttraumatic stress symptoms may avoid triggers like participation in our study and have a particular high risk of interventions during delivery. On the other hand, simply answering our questionnaire could be considered an intervention in itself. Recalling abuse when completing the questionnaire may trigger fear of childbirth and symptoms of depression, which again may affect the mode of delivery; hence, the observed associations may be seen as results of the study as an intervention. However, if this were the case, we would have likely seen increases in the estimated associations in all types of abuse.

Another important strength of our study is that we used a previously validated instrument to measure abuse. The validity of the NorAQ compared to clinical interviews and other instruments has been shown to be high [20]. Still women may have opted not to report abuse. This may have biased the estimated association towards zero. As this is a longitudinal study, a recall bias based on outcome is unlikely. Our study did not include questions on the onset, length of time and frequency of the abuse, nor who the perpetrator was. The influence of these factors could therefore not be studied. Our findings are based upon women from selected European countries, and may not be generalized to other locations with very different health care system.

Some of our findings are in conflict with other studies. A cohort study from Trondheim found that childhood abuse, whether it was physical or sexual, was associated with an increased risk of interventions during childbirth, both CS and instrumental vaginal delivery [13]. This study made no distinction between primiparous and multiparous women [13]. However, the lack of associations between childhood abuse and operative delivery agrees with the findings of a large population based study from Norway, which found no association between childhood abuse and CS before labor and only a slight increase for CS during labour [14]. The lack of association between childhood abuse and mode of delivery in our study could be due to low power in our study due to few cases. Alternatively, it may be the result of effective psychosocial counselling. An increased recognition and optimal intervention for depression and fear of delivery may prevent interventions. A Norwegian study including women attending a specialised clinic aimed at caring for women with fear of childbirth, reported that sexual abuse in the form of rape after the age of 16 years was associated with a major increase in risk for CS [15]. This is in agreement with our results. However, theirs was a selected population with whom mode of delivery was actively discussed as part of the consultation and hence the association may have been the consequence of an active choice during the consultation.

Sexual abuse is likely to affect pregnant women more than other types of abuse since the female reproductive organs are involved both in sexual acts and in giving birth [12], [28]. For this reason, the woman and/or the obstetrical staff may be anxious about a vaginal delivery, perhaps more so than for a history of physical or emotional abuse. An obstetrician who is aware of such a history may be more inclined to grant a CS on maternal request without any other medical indication, as our observed association indicate. In a situation where there is uncertainty about mode of delivery, knowledge of a woman's history of abuse and/or her wish for CS may influence the decision towards a CS with a medical indication. In our study, we had no knowledge about whether women revealed their history and/or preference for CS at any stage before delivery. There is no consensus about the optimal mode of delivery for women with a traumatic history of adult sexual abuse, nor do we know how alternative approaches may affect the mother-infant relationship and the long-term psychological effects of delivery/childbirth. However, our results suggest that efforts should be made to reduce suffering from the experienced abuse as current suffering increased the risk of not only elective but also emergency CS.

Women with a previous delivery and a history of abuse did not have an increased risk of operative delivery associated with the abuse. A possible explanation could be that victims of adult sexual abuse who either had an operative delivery and/or suffered a poor birth experience chose not to become pregnant again and hence they were not among the multiparous women in our study. It is also possible that the previous birth experience was a healing experience as described by Simpkin and Klaus [29].

An obvious possible reason for an increase in emergency CS among women reporting physical abuse would be placental abruption after trauma. This indication was not among the options in the outcome sheet in our study. The most common CS indications for these women were fetal distress and other medical indications, which could be related to direct physical trauma to the abdomen. However, when we added previous CS into the analysis, the association was clearly attenuated, indicating that this factor played a major role. The CS in the previous pregnancy could have been due to abuse prior to that. Our results indicated that a history of abuse has limited impact on mode of delivery for women with a previous birth experience. As in obstetrics in general, the method of the first birth has a great influence on subsequent mode of delivery [30].

## Conclusions

Among primiparous women sexual abuse experienced as an adult was associated with increased elective caesarean sections. Primiparous women with a history of adult sexual abuse were also more likely to have a non-obstetrically indicated CS. Identifying a woman's sexual abuse status during pregnancy may influence decision-making regarding mode of delivery.
